# UNDERSTANDING THE IMPACT OF AGEISM AMONG HEALTHCARE PROFESSIONALS AND STUDENTS

**DOI:** 10.1093/geroni/igad104.3681

**Published:** 2023-12-21

**Authors:** Laura Finn, Kavitha Sukumar, Serena Fasola, Dennis Phan, Kirsten Bautista, Michelle Hon, Catherine Oswald

**Affiliations:** Philadelphia College of Pharmacy / St Joseph’s University, Philadelphia, Pennsylvania, United States; Philadelphia College of Pharmacy / St Joseph’s University, Philadelphia, Pennsylvania, United States; Philadelphia College of Pharmacy / St Joseph’s University, Philadelphia, Pennsylvania, United States; Philadelphia College of Pharmacy / St Joseph’s University, Philadelphia, Pennsylvania, United States; Philadelphia College of Pharmacy / St Joseph’s University, Philadelphia, Pennsylvania, United States; Roseman University of Health Sciences, Henderson, Nevada, United States; Roseman University of Health Sciences, Henderson, Nevada, United States

## Abstract

With our population aging, the need for healthcare practitioners trained in caring for older adults is urgent. The anticipated shortage of geriatric-trained providers may further the impact of age-related discrimination. Little data is published about the understanding of ageism by healthcare professionals and students. The comparison of these different groups became the focus of the conducted survey, and the presenting research analyzed a total of 467 respondents including dental, nursing, pharmacy, occupational therapy, and physical therapy professions across 2 universities. When comparing demographic differences in studied populations, 69% of faculty/preceptors and 12% of students were over 35 years old. Overall, 33.8% reported being unable to define ageism with 27.7% of all students unable to do so. In looking specifically at pharmacy, 34.3% of students and 37.9% of faculty were unable to define ageism. Understanding ageism and its effects on healthcare for older adults is important for pharmacists as 82.1% of pharmacy faculty reported regularly interacting with older adults and 67% said that primarily occurred at work; yet only 37.5% had taken a class or course in geriatrics. Among pharmacy students, 71.4% interacted regularly with older adults with 41.4% saying this occurred primarily at work. Differences were noted in student education with 50% of pharmacy and 86.9% of physical therapy students having taken a geriatric course. Analysis and conclusions interpret the impact of ageism and emphasize a need for education to address age-related disparities and potential for bias in healthcare.

